# Phage WO diversity and evolutionary forces associated with *Wolbachia*-infected crickets

**DOI:** 10.3389/fmicb.2024.1499315

**Published:** 2025-01-08

**Authors:** Qing-Chen Luo, Yue-Yuan Li, Ye-Song Ren, Xiao-Hui Yang, Dao-Hong Zhu

**Affiliations:** ^1^Laboratory of Insect Behavior and Evolutionary Ecology, College of Life Science and Technology, Central South University of Forestry and Technology, Changsha, China; ^2^College of Life Science, Hunan Normal University, Changsha, China; ^3^College of Forestry, Central South University of Forestry and Technology, Changsha, China

**Keywords:** phage WO, multiple infections, recombination, horizontal transfer, *Wolbachia*, cricket

## Abstract

**Introduction:**

Phage WO represents the sole bacteriophage identified to infect *Wolbachia*, exerting a range of impacts on the ecological dynamics and evolutionary trajectories of its host. Given the extensive prevalence of *Wolbachia* across various species, phage WO is likely among the most prolific phage lineages within arthropod populations. To examine the diversity and evolutionary dynamics of phage WO, we conducted a screening for the presence of phage WO in *Wolbachia*-infected cricket species from China.

**Methods:**

The presence of phage WO was detected using a PCR-based methodology. To elucidate the evolutionary forces driving phage WO diversity, analyses of intragenic recombination were conducted employing established recombination techniques, and horizontal transmission was investigated through comparative phylogenetic analysis of the phages and their hosts.

**Results and discussion:**

Out of 19 cricket species infected with *Wolbachia*, 18 species were found to harbor phage WO. Notably, 13 of these 18 cricket species hosted multiple phage types, with the number of types ranging from two to 10, while the remaining five species harbored a single phage type. Twelve horizontal transmission events of phage WO were identified, wherein common phage WO types were shared among different *Wolbachia* strains. Notably, each phage WO horizontal transfer event was associated with distinct *Wolbachia* supergroups, specifically supergroups A, B, and F. Previous studies have found that four *Wolbachia* strains infect two to five species of crickets. However, among these cricket species, in addition to the shared phage WO types, all harbored species-specific phage WO types. This suggests that *Wolbachia* in crickets may acquire phage WO types through horizontal viral transfer between eukaryotes, independent of *Wolbachia* involvement. Furthermore, nine putative recombination events were identified across seven cricket species harboring multiple phage types. These findings suggest that horizontal transmission and intragenic recombination have played a significant role in the evolution of the phage WO genome, effectively enhancing the diversity of phage WO associated with crickets.

## Introduction

*Wolbachia*, members of the Anaplasmataceae family, are maternally inherited endosymbiotic bacteria that infect arthropods and filarial nematodes (Werren et al., 2008; Engelstädter and Hurst, 2009). These bacteria manipulate their host's reproduction by inducing various phenotypes, including cytoplasmic incompatibility (CI), parthenogenesis, feminization of genetic males, and male-killing (Werren et al., 2008).

Bacteriophages, the most abundant entities on Earth, play a pivotal role in the evolution of bacterial genomes (Hendrix et al., 1999; Bordenstein and Wernegreen, 2004). *Wolbachia* phage, a λ phage-like temperate double-stranded DNA (dsDNA) bacteriophage known as phage WO, was initially characterized from the *Wolbachia* strain wTai, which infects the cricket species *Teleogryllus taiwanemma*. Phage WO can exist in either a lysogenic state, integrated into the *Wolbachia* chromosome, or a lytic state, free in the cytoplasm (Masui et al., 2000, 2001). It is estimated that phage WO infects ~90% of *Wolbachia* supergroups A and B across various arthropod groups, but it is absent in the mutualistic C and D supergroups typically found in filarial nematodes (Bordenstein and Wernegreen, 2004; Gavotte et al., 2007; Gerth et al., 2014). Bordenstein and Bordenstein (2022) proposed that all phage WO should be classified within the genus *Wovirus*, belonging to the family Symbioviridae. Given the persistence of phage WO, it is hypothesized that phage WO confers certain advantages to its *Wolbachia* and arthropod hosts (Kent and Bordenstein, 2010). The transformation and integration of prophages are regarded as the primary mechanisms of lateral gene acquisition in *Wolbachia*, analogous to other prokaryotes (Bordenstein et al., 2006). Phage WO may facilitate lateral gene transfer in *Wolbachia* strains (Ishmael et al., 2009; Wang et al., 2016) and modulate *Wolbachia* density by inducing cell lysis or inhibiting replication (Bordenstein et al., 2006). Recent studies have identified the factors underlying CI as two genes, *cifA* and *cifB*, which are situated adjacent to one another within WO prophage regions of the *Wolbachia* genome. These genes have homologs in all known CI-inducing *Wolbachia* strains (Beckmann et al., 2017; LePage et al., 2017; Chen et al., 2019; Shropshire and Bordenstein, 2019).

The survey results of Gavotte et al. (2007) and Tanaka et al. (2009) indicated that the majority of phage-infected *Wolbachia* strains harbor a limited number of phage types, with ~85% containing only one or two distinct phage types. However, accumulating evidence suggests the occurrence of multiple phage infections in certain *Wolbachia* strains, with some strains exhibiting more than two phage types (Chauvatcharin et al., 2006; Gavotte et al., 2007; Zhu et al., 2021; Gao et al., 2022). For instance, 84% of the 19 butterflies collected from China, each infected with a single *Wolbachia* strain, were found to contain high levels of multiple phage types, ranging from 3 to 17 types (Gao et al., 2022). Additionally, Zhu et al. (2021) confirmed that the *Wolbachia*-infected gall wasp *Andricus hakonensis* harbored 27 distinct phage WO types.

Mutation, recombination, and genome segment reassortment are potential mediators of genetic alterations in viruses during replication (Domingo, 2010). The genome of a bacteriophage can be partitioned into functional units, or modules, each responsible for distinct processes such as head or tail formation, lysis, lysogeny, among others. Furthermore, within the shared gene pool, the primary evolutionary mechanism driving changes in double-stranded DNA bacteriophages is the rearrangement of genomic fragments through interactions with other bacteriophages (Hatfull, 2008). Phage WO is capable of evolving via mechanisms such as point mutation, intragenic recombination, deletion, and purifying selection (Kent et al., 2011). The nucleotide sequence of the minor capsid gene *orf7* in the wKueA1 strain of *Wolbachia* exhibits a chimeric nature, with multiple intragenic recombination events identified through population genetic analysis (Bordenstein and Wernegreen, 2004). Previous research has offered molecular evidence of recombination within the *orf7* gene, indicating that intragenic recombination serves as a significant evolutionary mechanism that substantially enhances the diversity of phage WO in gall wasps (Zhu et al., 2021) and butterflies (Gao et al., 2022). Additionally, base deletions occurring during replication have been shown to significantly drive the evolution of the phage genome, contributing to the diversity of phage WO associated with *A. hakonensis* (Su et al., 2021).

Numerous studies have identified *Wolbachia* infections in crickets. For instance, Giordano et al. (1997) documented the presence of *Wolbachia* in *Gryllus assimilis, G. ovisopis, G. integer, G. rubens*, and *G. pennsylvanicus*. In a significant contribution from China, Li et al. (2022) conducted the first comprehensive analyses of *Wolbachia* in crickets, revealing that 19 species across eight genera tested positive for the infection. Furthermore, Kamoda et al. (2000) confirmed the association between CI and *Wolbachia* infection in the cricket species *Teleogryllus taiwanemma*. *Wolbachia* may induce incomplete CI and enhance female fertility in *Velarifictorus aspersus* (Zhu and Liu, 2024). Additionally, Chafee et al. (2010) analyzed phage genes from natural sympatric field isolates in *Gryllus pennsylvanicus*. Kupritz et al. (2021) reported the isolation and characterization of a novel phage WO from *Allonemobius socius*. However, prior investigations into phage WO infections of *Wolbachia* in Chinese crickets have been lacking. Consequently, this study screened 19 species of *Wolbachia*-infected crickets for the presence of phage WO utilizing a PCR-based methodology. Additionally, to elucidate the evolutionary forces of phage WO diversity, we conducted analyses of intragenic recombination through established recombination techniques and examined horizontal transmission via comparative phylogenetic analysis of the phages and their hosts.

## Materials and methods

### Insect and DNA extraction

We used published DNA samples in this study. Insects collection, DNA extraction, and *Wolbachia* amplification using multi-locus sequence typing (MLST) gene primers have been described in Li et al. (2022).

### PCR and sequencing

Total DNA was extracted from each cricket individual. Phage WO infections were detected individually by using PCR to amplify a segment of the capsid protein gene *orf7* with the primers WO-F (5′-CCCACATGAGCCAATGACGTCTG-3′) and WO-R (5′- CGTTCGCTCTGCAAGTAACTCCATTAAAAC-3′) (Masui et al., 2000). PCR amplification was carried out using a C1000 Touch thermal cycler (Bio-Rad, Hercules, CA, United States) in a reaction volume of 25 μL, consisting of 18.375 μL ddH_2_O, 2.5 μL PCR buffer, 2 μL dNTPs (10 mmol each), 1 μL of both forward and reverse primers (10 mM), 1 μL DNA extract, and 0.125 μL Taq polymerase (Takara, Dalian, China). The PCR cycling parameters included an initial denaturation step at 95°C for 3 min, followed by 35 cycles at 95°C for 30 s, 57°C for 40 s, and 72°C for 40 s, with a final extension step at 72°C for 5 min.

The *orf7* fragment of each cricket species was sequenced from two to three individuals, with subsequent purification of the PCR products and direct sequencing of the *orf7* gene fragments in both directions using PCR primers. The presence of multiple peaks during initial sequencing of each sample was interpreted as indicative of multiple infections. The PCR products underwent purification through the utilization of a DNA gene gel extraction kit and subsequent ligation into the pMD18-T cloning vector in adherence to the manufacturer's guidelines. Following this, 15 distinct positive colonies were isolated from each sample and cultivated in lysogeny broth medium containing ampicillin (100 mg/mL) and d-biotin (2 mM). Plasmids were then extracted and subjected to sequencing in both forward and reverse directions using M13F/R primers on an ABI 3730XL DNA sequencer (Applied Biosystems, Foster City, CA, United States).

### Phage WO typing and phylogenetic analysis

Sequence analysis and homology comparisons were conducted utilizing the online BLAST program. Genetic distances between all sequence pairs were determined using the Kimura two-parameter distance model with the complete deletion option in MEGA software (version 4.0). Sequences exhibiting genetic distances below 1.5% nucleotide diversity in the *orf7* gene were classified as identical haplotypes, as outlined by Chafee et al. (2010) and Zhu et al. (2021). The sequences have been deposited in GenBank under the following accession numbers: PQ674069–674193. The sequences of *Wolbachia* MLST genes (*gatB, coxA, hcpA, fbpA*, and *ftsZ*, were retrieved from Li et al., 2022, corresponding to GenBank MW680307–6803334) and the phage WO *orf7* gene were aligned, respectively, using SEQMAN PRO v.11.2 (DNASTAR, Madison, WI, USA), followed by analysis using the neighbor joining method in PAUP v.4.0b (Swofford, 2003). The best evolutionary model was selected according to the corrected Akaike information criterion calculated using MEGA v.7.0. Bootstrap tests were conducted based on 1,000 replicates to assess branch support in the final maximum likelihood trees.

### Recombination analysis

The individual segment alignments were examined for signs of intragenic recombination through various techniques within the Recombination Detection Program (RDP5) package (Heath et al., 2006). Six recombination detection methods available in the RDP5 program, including BootScan/rescan recombination test (Martin et al., 2005), 3Seq (Martin and Rybicki, 2000), Chimera (Posada and Crandall, 2001), GENECONV (Padidam et al., 1999), Siscan method (Gibbs et al., 2000), and MaxChi (Smith, 1992), were employed to identify recombinant sequences and breakpoints. Default settings were applied to all methods, with a maximum acceptable *P*-value cutoff of 0.05.

## Results

### Phage WO infections

The reanalysis of *Wolbachia* MLST sequences from Li et al. (2022) allowed to identify 15 distinct sequence types (STs) belonging to supergroups A, B, and F in 19 out of the 22 cricket species under study. Phage WO was identified by PCR in 18 out of the 19 *Wolbachia*-infected cricket species (*T. mitratus* species did not show any positive *orf7* PCR), rendering 127 phage WO *orf7* sequences (see Table 1; Supplementary Table 1). Phage WO types with *orf7* DNA sequences demonstrating a similarity > 98.5% were classified as identical types based on previous research (Chafee et al., 2010; Zhu et al., 2021). Each phage type was labeled as WO followed by the insect name and haplotype number. For instance, *T. infernalis* contained a singular phage type designated as WOTin, while *Loxoblemmus taicoun* from a specific geographical population in Changsha possessed two distinct phage types labeled as WOLta-1-CS and WOLta-2-CS. Thirteen *Wolbachia*-infected cricket species hosted multiple phage types and the other species harbored one type (Table 1; Supplementary Figures 1–3).

**Table 1:** *Wolbachia* strains and phage WO types in Gryllidae.

**Host species**		***Wolbachia*** strains^*^			**WO type number**
**Species**	**Location**	**ST** ^†^	***wsp*** **allele (accession no.)**	**Supergroup**	
*Velarifictorus micado*	TA	b	MW680307	B	9
	CS	k	MW680308	F	10
	ZJJ	j	MW680309	F	8
	LL	a	MW680310	B	5
*Velarifictorus asperses*	ZJ	f	MW680311	F	4
*Velarifictorus khasiensis*	JS	j	MW680312	F	6
*Teleogryllus emma*	ZJ	N/A	MW680313	A	2
*Teleogryllus occipitalis*	ZJ	32	MW680314	B	2
*Teleogryllus infernalis*	ES	i	MW680315	F	1
*Teleogryllus mitratus*	ZJ	k	MW680332	F	0
*Loxoblemmus* sp1.-1	TA	d	MW680316	B	5
*Loxoblemmus* sp1.-2	TA	l	MW680317	A	1
*Loxoblemmus* sp2.	TA	l	MW680318	A	1
*Loxoblemmus* sp3.	CS	543	MW680319	B	2
*Loxoblemmus* sp4.	JS	k	MW680333	F	1
*Loxoblemmus angulatus*	ZJ	245	MW680320	F	1
*Loxoblemmus doenitzi*	TA	l	MW680321	A	3
	TA-2	N/A	MW680334	F	0
	LL	c	MW680322	B	7
*Loxoblemmus taicoun*	LL	l	MW680323	A	1
	JS	543	MW680324	B	2
*Loxoblemmus jacobson*i	JS	k	MW680325	F	3
*Loxoblemmus montanus*	CZ	543	MW680326	B	6
*Mitius minor*	JS	l	MW680327	F	8
*Dianemobius* sp.	ZJ	e	MW680328	B	1
*Polionemobius taprobanensis*	JS	g	MW680329	B	1
*Comidoblemmus nipponensis*	CZ-1	h	MW680330	B	6
	CZ-2	N/A	MW680331	A	1

^*^Data from Li et al. (2022). N/A indicates no data available.

^†^ST refers to multi-locus sequence types.

### Phage WO diversity in *Wolbachia* strains

**Figure 1:**
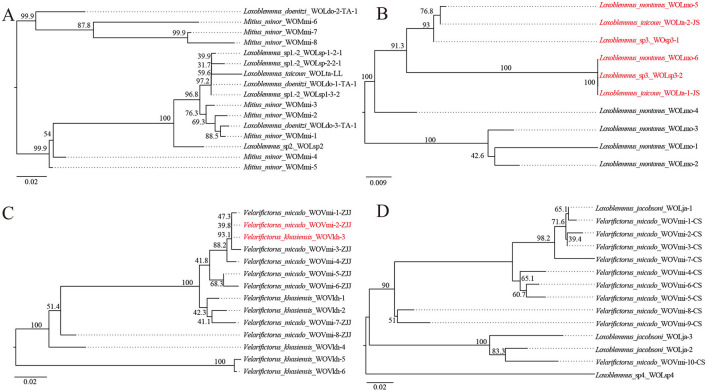
Neighbor joining phylogenetic tree obtained based on phage WO *orf7* nucleotide sequences from *Wolbachia* strains ST-l **(A)**, ST-543 **(B)**, ST-j **(C)**, and ST-k **(D)**. Numbers above branches are bootstrap values based on 1,000 replicates. WO-1 refers to the serial number. Red font indicates identical *orf7* sequences or those with similarity >98.5% to their respective sequences.

Previous research has identified that four strains of *Wolbachia*—ST-l (supergroup A), ST-543 (supergroup B), ST-j, and ST-k (supergroup F)—infect between two to five species of crickets (Li et al., 2022) (Table 1). To investigate the prevalence of phage WO infections within these *Wolbachia* strains, phylogenetic trees were constructed based on the *orf7* sequences of phage WO types that infected *Wolbachia* strains across various insect species (Figure 1). *Wolbachia* strain ST-l was identified in five cricket species, including *L. doenitzi, Loxoblemmus* sp1., *Loxoblemmus* sp2., *L. taicoun*, and *Mitius minor*, while ST-k was present in four cricket species, namely *Teleogryllus mitratus, L. jacobsoni, Velarifictorus micado*, and *Loxoblemmus* sp4. *Wolbachia* strains ST-l and ST-k were found to be infected with 16 and 14 phage WO types, respectively, with no identical phage types observed among different cricket species (Figures 1A, D). *Wolbachia* strain ST-543 was identified in *L. taicoun, L. montanus*, and *Loxoblemmus* sp3, with these cricket species sharing two common phage WO types. Specifically, the sequences of WOLmo-6, WOLsp3-2, and WOLta-1-JS were identical, while those of WOLmo-5, WOLta-2-JS, and WOsp3-1 either matched or contained one base substitution. The remaining four phage WO types were exclusively present in *L. montanus* (Figure 1B). *Wolbachia* strain ST-j was found in *V. micado* and *V. khasiensis*. These two species of *Velarifictorus* shared a common phage WO type, as evidenced by the identical sequences of WOVmi-2-ZJJ and WOVkh-3 (Figure 1C).

### Horizontal transfer

**Figure 2:**
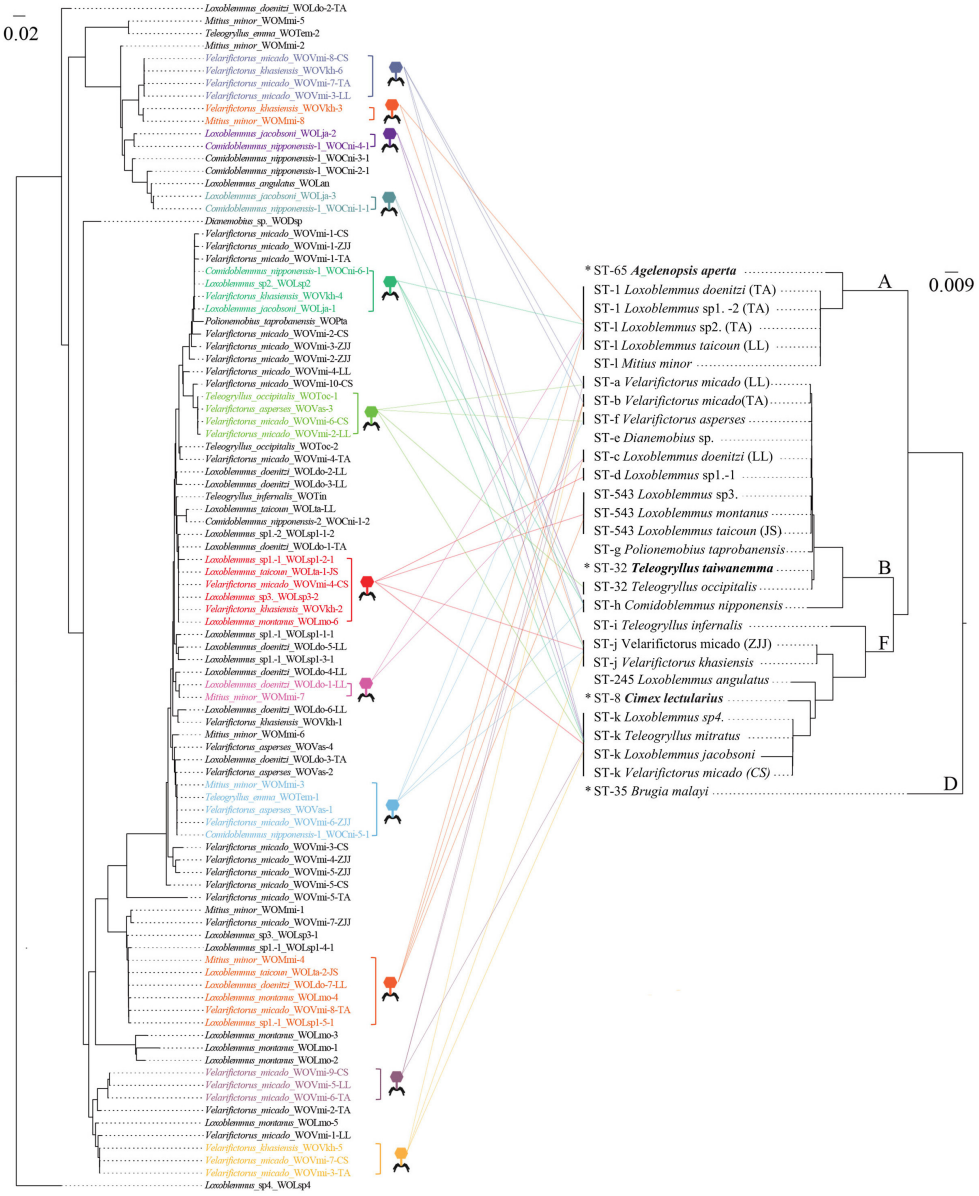
Comparisons of neighbor joining phylogenetic tree obtained for phage WO based on *orf7* nucleotide sequences **(Left)** and *Wolbachia* based on concatenated sequences of multi-locus sequence type (MLST) genes (from Li et al., 2022) **(Right)**. Colored fonts indicate identical *orf7* sequences or those with similarity >98.5% to their respective sequences. The latter sequences are shown by icons with different colors and each icon represents a horizontal transfer event. The capital letters on the right indicate the *Wolbachia* supergroups. *Refers to known *Wolbachia* symbionts of various crickets retrieved from the MLST database.

Phylogenetic analysis was performed on phage WO *orf7* sequences and concatenated sequences of five *Wolbachia* MLST genes from crickets using neighbor joining methods (Figure 2). The results of the analysis revealed a lack of congruence between the phylogenies of phage WO and its host *Wolbachia*. Additionally, evidence was found for 12 instances of horizontal transmission of phage WO from crickets, where different *Wolbachia* strains shared common phage WO types; six involved four *Wolbachia* strains, four involved two *Wolbachia* strains, and one involved three *Wolbachia* strains (Figure 2). For instance, WOVmi-8-CS (derived from *V. micado*-CS), WOVkh-6 (derived from *V. khasiensis*), WOVmi-7-TA (derived from *V. micado*-TA), and WOVmi-3-LL (derived from *V. micado*-LL) exhibited identical *orf7* sequences, indicating their classification within the same WO phage type. These phages were found to infect a minimum of four *Wolbachia* strains, including ST-a, ST-b, ST-j, and ST-k. The *Wolbachia* strains present in *T. emma, L. doenitzi*-TA-2, and *Comidoblemmus nipponensis*-CZ-2 could not be fully characterized due to difficulties in amplifying certain MLST loci (Li et al., 2022). All 12 instances of horizontal transmission involved distinct *Wolbachia* supergroups, with two instances occurring between supergroups A and B, one instance occurring between supergroups A and F, seven instances occurring between supergroups B and F, and two instances involving supergroups A, B, and F simultaneously.

### Intragenic recombination

To ascertain direct evidence of phage WO intragenic recombination, recombination analysis was conducted on aligned *orf7* sequences sourced from the same cricket species utilizing the RDP5 software. Recombination events were exclusively included if identified by at least three detection methods within RDP5.

**Figure 3:**
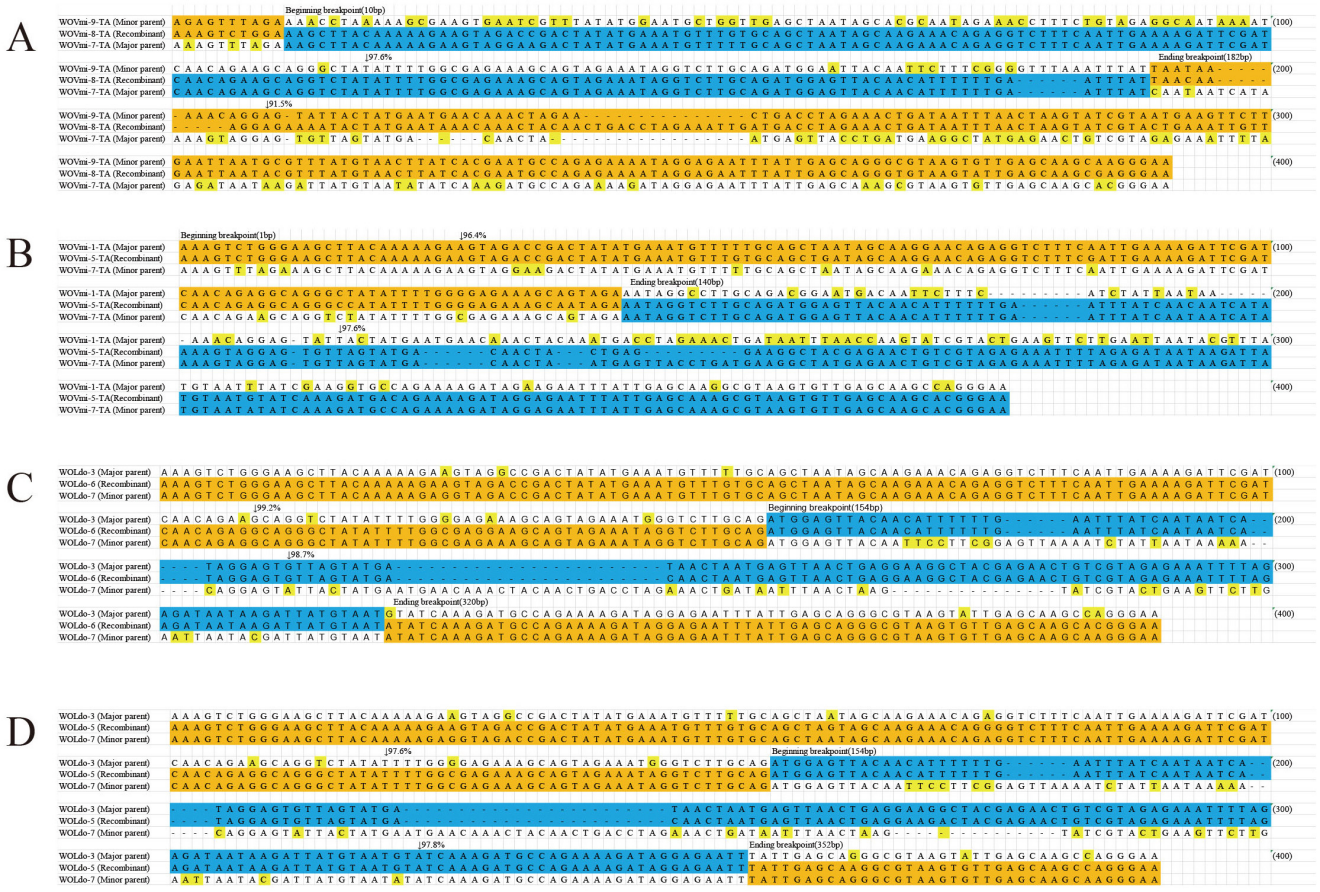
Recombination events identified for the *orf7* gene between: WOVim-9-TA and WOVim-7-TA resulting in recombinant WOVim-8-TA **(A)**; WOVim-1-TA and WOVim-7-TA resulting in recombinant WOVim-5-TA **(B)**; WOLdo-3 and WOLdo-7 resulting in recombinant WOLdo-6 **(C)**; and WOLdo-3 and WOLdo-7 resulting in recombinant WOLdo-5 **(D)**.

Nine putative recombination events were identified among seven cricket species that contained a variety of phage WO types (Table 2; Figure 3; Supplementary Figures 4, 5). These events involved a major and minor parent, both of which recombined at the same breakpoint to form a new phage WO lineage (Table 2). For instance, the phage WO type WOVmi-8-TA was found to be a recombinant using five of the six methods employed: 3Seq (*P* < 10^−12^), BootScan (*P* < 10^−6^), GENECONV (*P* < 10^−5^), Chimera (*P* < 10^−7^), and MaxChi (*P* < 10^−9^). The parental genotypes identified in this study were designated as WOVmi-7-TA and WOVmi-9-TA for the major and minor parents, respectively, with breakpoints located at positions 10 and 182 bp (Figure 3A). Furthermore, it was observed that a major parent in one recombinant could serve as a minor parent in another recombinant. For instance, WOVmi-7-TA acted as the major parent in the formation of recombinant WOVmi-8-TA, while simultaneously serving as the minor parent in the generation of WOVmi-5-TA (Figure 3B). It is important to acknowledge that major and minor parents have the ability to recombine through different breakpoints, resulting in the production of various recombinants. For instance, the recombinant WOLdo-6 and WOLdo-5 originated from the major parent WOLdo-3 and minor parent WOLdo-7, with breakpoints at 320 and 325 bp, respectively (Figures 3C, D). These findings indicate that intragenic recombination is prevalent and frequent in the phage WO types found in crickets.

**Table 2:** Recombination analysis based on the phage WO *orf7* gene in Gryllidae using six methods implemented in the RDP5 package.

**Insect**	**Recombinant**	**Major parent**	**Minor parent**	**Breakpoint**	**Method**	***P*-value**
*Velarifictorus micado* (TA)	WOVmi-8-TA	WOVmi-7-TA	WOVmi-9-TA	10/182	GENECONV	6.52E-06
					BootScan	7.01E-07
					MaxChi	2.44E-10
					Chimera	1.52E-08
					3Seq	8.39E-13
*Velarifictorus micado* (TA)	WOVmi-5-TA	WOVmi-1-TA	WOVmi-7-TA	1/140	BootScan	1.80E-03
					MaxChi	4.76E-04
					Chimera	1.44E-02
					3Seq	1.84E-07
*Velarifictorus micado* (CS)	WOVmi-8-CS	WOVmi-4-CS	WOVmi-10-CS	102/300	GENECONV	1.13E-07
					BootScan	7.11E-09
					MaxChi	1.09E-11
					Chimera	3.83E-12
					3Seq	2.17E-11
*Velarifictorus micado* (LL)	WOVmi-1-LL (WOS-2)	WOVmi-1-LL (WOS-1)	WOVmi-3-LL	102	GENECONV	1.02E-07
					BootScan	2.08E-07
					MaxChi	6.82E-11
					Chimera	6.96E-12
					3Seq	9.70E-10
*Velarifictorus khasiensis*	WOKha-4	WOKha-2	WOKha-6	164/317	GENECONV	1.44E-06
					BootScan	2.65E-07
					MaxChi	4.37E-09
					Chimera	9.80E-10
					SiSscan	4.97E-12
					3Seq	1.95E-02
*Loxoblemmus doenitzi* (LL)	WOLdo-6	WOLdo-3	WOLdo-7	154/320	BootScan	5.14E-03
					MaxChi	5.51E-03
					Chimera	4.72E-02
					3Seq	2.55E-05
*Loxoblemmus doenitzi* (LL)	WOLdo-5	WOLdo-3	WOLdo-7	154/325	BootScan	1.60E-02
					MaxChi	1.70E-02
					3Seq	5.84E-03
*Mitius minor*	WOMmi-4	WOMmi-2	WOMmi-6	162/301	RDP	1.51E-07
					GENECONV	7.53E-06
					BootScan	1.53E-07
					MaxChi	1.86E-08
					Chimera	1.09E-08
					SiSscan	1.72E-06
					3Seq	2.31E-10
*Comidoblemmus nipponensis*	WOCni-3-1	WOCni-1-1	WOCni-4-1	120/276	BootScan	0.021
					MaxChi	2.66E-02
					3Seq	1.05E-03

## Discussion

Previous studies have demonstrated the occurrence of *Wolbachia* infections in various cricket genera, including *Gryllus, Velarifictorus, Teleogryllus, Allonemobius, Loxoblemmus, Mitius, Dianemobius, Gryllodes, Polionemobius*, and *Comidoblemmus* (Giordano et al., 1997; Kamoda et al., 2000; Mandel et al., 2001; Marshall, 2004; Jeong et al., 2012; Li et al., 2022). *Wolbachia* may induces CI in several cricket species, such as *Teleogryllus taiwanemma* (Kamoda et al., 2000) and *Velarifictorus aspersus* (Zhu and Liu, 2024). Nevertheless, the investigation of phage WO, which is associated with *Wolbachia*-infected crickets, has been limited to a relatively small number of studies (Masui et al., 2000; Chafee et al., 2010; Kupritz et al., 2021). In the present study, we report the first detection of phage WO infections associated with crickets in China. Our findings reveal that among the 19 *Wolbachia*-infected cricket species examined, phage WO was present in 18 species spanning the genera *Velarifictorus, Teleogryllus, Loxoblemmus, Mitius, Dianemobius, Polionemobius*, and *Comidoblemmus*, indicating a high prevalence of infection. Most *Wolbachia* strains infected by phage WO exhibited a limited diversity of phage types, with 85% containing only one or two distinct phage types (Gavotte et al., 2007; Tanaka et al., 2009). Nevertheless, instances of multiple phage WO infections have been documented in various *Wolbachia* strains (Gavotte et al., 2007; Zhu et al., 2021; Gao et al., 2022). Our findings revealed that 13 cricket species infected with *Wolbachia* harbored multiple phage types, with six of these species carrying more than five distinct phage types. For example, the Changsha population of *V. micado* infected with *Wolbachia* strain ST-k harbored 10 phage WO types, and *V. khasiensis* infected with *Wolbachia* strain ST-j harbored six phage WO types. These results indicate that *Wolbachia*-infected cricket species possess a high diversity of phage WO types, consistent with observations in gall wasps (Zhu et al., 2021) and butterflies (Gao et al., 2022).

Phage WO can disseminate among hosts through both vertical and horizontal transmissions. The absence of significant correlations between the evolutionary phylogenies of WO and *Wolbachia* (Bordenstein and Wernegreen, 2004; Gavotte et al., 2004; Zhu et al., 2021; Gao et al., 2022), along with the presence of divergent *Wolbachia* strains infecting either the same (Gavotte et al., 2004; Chauvatcharin et al., 2006) or different hosts (Wang et al., 2016; Zhu et al., 2021; Gao et al., 2022) that harbor identical phage WO types, indicates that numerous horizontal transfers of phage WO have likely occurred among various *Wolbachia* strains. In our study, we identified twelve instances of horizontal phage WO transmission, wherein distinct *Wolbachia* strains in crickets exhibited shared phage WO types. Remarkably, each of these horizontal transfer events was linked to different *Wolbachia* supergroups, specifically supergroups A, B, and F. During the lytic phase, prophages have the capacity to disrupt the cell membranes of both bacterial and eukaryotic cells. Subsequently, phage WO resides within the extracellular matrix of arthropods, enabling it to traverse the eukaryotic cell wall and initiate new infections (Masui et al., 2001; Bordenstein et al., 2006; Gavotte et al., 2007). *Wolbachia* strains ST-l, ST-543, ST-j, and ST-k were found to be shared among two to five cricket species (Li et al., 2022). Apart from the common phage WO types, nearly all of the cricket species harbored distinct, species-specific phage WO types. These findings strongly suggest that *Wolbachia* in crickets may acquire phage WO types through horizontal viral transfer between eukaryotes, independent of *Wolbachia* involvement, as has been previously reported in butterflies (Gao et al., 2022). Therefore, the horizontal transmission of phage WO—encompassing interactions across various supergroups of *Wolbachia* and among different insect hosts—may significantly contribute to the diversification of phage WO within *Wolbachia*-infected crickets.

Recombination and reassortment are recognized as mechanisms that enable RNA and DNA viruses to adapt to fluctuating environments. These processes can enhance viral genetic diversity and virulence, potentially leading to an expansion of host range (Domingo, 2010; Franzo et al., 2016; Chen et al., 2018). In the case of phage WO, the capsid protein gene *orf7* undergoes frequent recombination, which substantially contributes to the genetic diversity of phage WO associated with *Wolbachia*-infected gall wasps (Zhu et al., 2021) and butterflies (Gao et al., 2022). Our research revealed that certain crickets were infected with a single *Wolbachia* strain harboring multiple phages. We identified nine recombination events within cricket species harboring diverse phage WO types. In certain phage WO lineages, a major parent in one recombinant could act as a mini parent in another recombinant; major and minor parental strains possess the capacity to recombine at different breakpoints and leading to the generation of various recombinant forms, thereby promoting active and frequent recombination. These findings indicate that intragenic recombination is a crucial evolutionary mechanism contributing to the high diversity of phage WO types associated with crickets.

In summary, 18 out of 19 cricket species infected with *Wolbachia* were observed to harbor phage WO, with 13 species hosting multiple phage types. This finding indicates a significant diversity of phage WO types associated with *Wolbachia*-infected crickets. Furthermore, twelve horizontal transmission events of phage WO were identified, and different *Wolbachia* strains within crickets shared common phage WO types. Additionally, nine recombination events were detected in cricket species harboring diverse phage WO types. These results suggest that intragenic recombination and horizontal transmission are pivotal evolutionary forces contributing to the observed diversity of phage WO in *Wolbachia*-infected crickets.

## Data Availability

The data presented in this study are deposited in the GeneBank repository, accession number PQ674069-674193.
