# Supplementary material for: Phage WO diversity and evolutionary forces associated with Wolbachia-infected crickets
Source: Front Microbiol. 2025 Jan 8;15:1499315. doi: 10.3389/fmicb.2024.1499315 (PMC11750818; doi:10.3389/fmicb.2024.1499315)
Supplement: Supplementary file 1 [file Table_1.docx]

Supplementary table 1 | Sample information and infection frequency of *Wolbachia* and Phage WO in Gryllidae.

| Location (code) | Latitude, longitude | Insect species | *Wolbachia* infect frequency (%)^*^ | Wo infect frequency (%) | WO type number | Individuals screened |
| --- | --- | --- | --- | --- | --- | --- |
| Taian, Shandong (TA) | 31°54′N, 114°89′E | *Velarifictorus micado* | 100 (single) | 100 | 9 | 23 |
| Changsha, Hunan (CS) | 28°13′N, 112°99′E | *Velarifictorus micado* | 100 (single) | 100 | 10 | 19 |
| Zhangjiajie, Hunan (ZJJ) | 29°24′N, 111°00′E | *Velarifictorus micado* | 100 (single) | 100 | 8 | 16 |
| Lvliang, Shanxi (LL) | 37°12′N, 111°78′E | *Velarifictorus micado* | 100 (single) | 100 | 5 | 35 |
| Zhanjiang, Guangdong (ZJ) | 21°38′N, 110°27′E | *Velarifictorus aspersus* | 100 (single) | 100 | 4 | 15 |
| Jishou, Hunan (JS) | 28°32′N, 109°74′E | *Velarifictorus khasiensis* | 100 (single) | 100 | 6 | 15 |
| Zhanjiang, Guangdong (ZJ) | 21°38′N, 110°27′E | *Teleogryllus emma* | 100 (single) | 100 | 2 | 2 |
| Zhanjiang, Guangdong (ZJ) | 21°38′N, 110°27′E | *Teleogryllus occipitalis* | 100 (single) | 100 | 2 | 3 |
| Eerduosi, Inner Mongolia (ES) | 39°61′N, 109°78′E | *Teleogryllus infernalis* | 100 (single) | 100 | 1 | 2 |
| Zhanjiang, Guangdong (ZJ) | 21°38′N, 110°27′E | *Teleogryllus mitratus* | 100 (single) | 100 | 0 | 1 |
| Taian, Shandong (TA) | 31°54′N, 114°89′E | *Loxoblemmus* sp1.-1 | 100 (single) | 100 | 5 | 10 |
| Taian, Shandong (TA) | 31°54′N, 114°89′E | *Loxoblemmus* sp1.-2 | 100 (single) | 100 | 1 | 10 |
| Taian, Shandong (TA) | 31°54′N, 114°89′E | *Loxoblemmus* sp2. | 100 (single) | 100 | 1 | 3 |
| Changsha, Hunan (CS) | 28°13′N, 112°99′E | *Loxoblemmus* sp3. | 100 (single) | 100 | 2 | 13 |
| Jishou, Hunan (JS) | 28°32′N, 109°74′E | *Loxoblemmus* sp4. | 12.5 (single) | 100 | 1 | 8 |
| Zhanjiang, Guangdong (ZJ) | 21°38′N, 110°27′E | *Loxoblemmus angulatus* | 100 (single) | 100 | 1 | 3 |
| Lvliang, Shanxi (LL) | 37°12′N, 111°78′E | *Loxoblemmus doenitzi* | 66.7 (single) | 100 | 3 | 6 |
| Taian, Shandong (TA) | 31°54′N, 114°89′E | *Loxoblemmus doenitzi* | 77.8 (multiple) | 50% | 3 | 9 |
| Zhangjiajie, Hunan (ZJJ) | 29°24′N, 111°00′E | *Loxoblemmus appendicularis* | 0 | – | – | 28 |
| Jishou, Hunan (JS) | 28°32′N, 109°74′E | *Loxoblemmus taicoun* | 100 (single) | 100 | 2 | 11 |
| Lvliang, Shanxi (LL) | 37°12′N, 111°78′E | *Loxoblemmus taicoun* | 100 (single) | 100 | 1 | 1 |
| Chenzhou, Hunan (CZ) | 26°06′N, 113°94′E | *Loxoblemmus montanus* | 40 (single) | 100 | 6 | 10 |
| Jishou, Hunan (JS) | 28°32′N, 109°74′E | *Loxoblemmus jacobsoni* | 100 (single) | 100 | 3 | 19 |
| Jishou, Hunan (JS) | 28°32′N, 109°74′E | *Mitius minor* | 70 (single) | 100 | 8 | 10 |
| Jishou, Hunan (JS) | 28°32′N, 109°74′E | *Dianemobius fascipes* | 0 | – | – | 10 |
| Zhanjiang, Guangdong (ZJ) | 21°38′N, 110°27′E | *Dianemobius* sp. | 20 (single) | 100 | 1 | 5 |
| Zhanjiang, Guangdong (ZJ) | 21°38′N, 110°27′E | *Gryllodes sigillatus* | 0 | – | – | 5 |
| Jishou, Hunan (JS) | 28°32′N, 109°74′E | *Polionemobius taprobanensis* | 100 (single) | 100 | 1 | 10 |
| Chenzhou, Hunan (CZ) | 26°06′N, 113°94′E | *Comidoblemmus nipponensis*-1 | 100 (single) | 100 | 6 | 16 |
| Chenzhou, Hunan (CZ) | 26°06′N, 113°94′E | *Comidoblemmus nipponensis*-2 | 100 (single) | 100 | 1 | 16 |

* Data from Li et al. (2022).
