# Supplementary material for: Phage WO diversity and evolutionary forces associated with Wolbachia-infected crickets
Source: Front Microbiol. 2025 Jan 8;15:1499315. doi: 10.3389/fmicb.2024.1499315 (PMC11750818; doi:10.3389/fmicb.2024.1499315)
Supplement: Supplementary file 2 [file Presentation_1.pptx]

## Slide 1
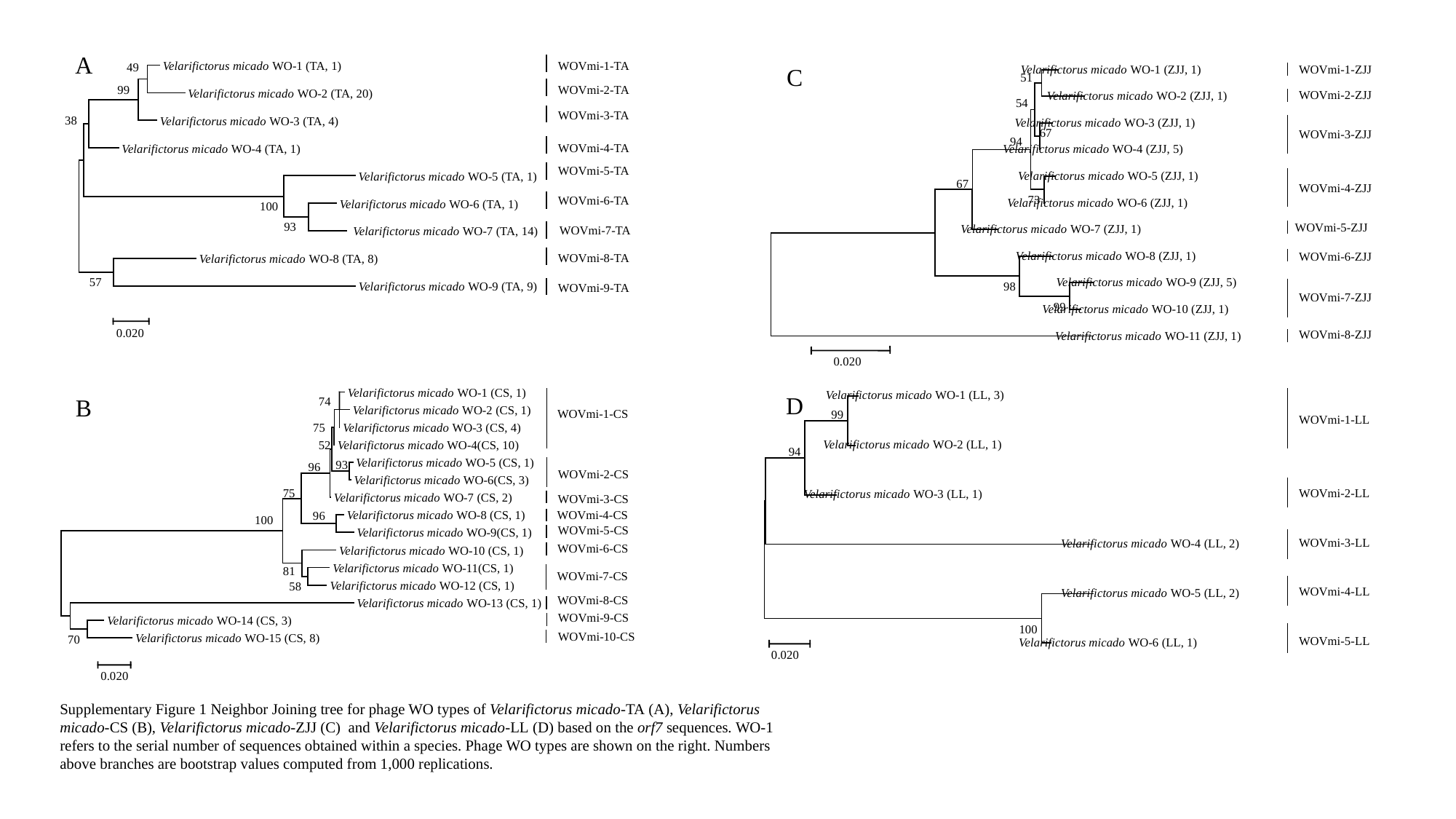

Velarifictorus micado WO-1 (TA, 1)
49
99
 Velarifictorus micado WO-2 (TA, 20)
38
 Velarifictorus micado WO-3 (TA, 4)
 Velarifictorus micado WO-4 (TA, 1)
 Velarifictorus micado WO-5 (TA, 1)
 Velarifictorus micado WO-6 (TA, 1)
100
93
 Velarifictorus micado WO-7 (TA, 14)
 Velarifictorus micado WO-8 (TA, 8)
57
 Velarifictorus micado WO-9 (TA, 9)
0.020
A
 Velarifictorus micado WO-1 (ZJJ, 1)
51
 Velarifictorus micado WO-2 (ZJJ, 1)
54
 Velarifictorus micado WO-3 (ZJJ, 1)
67
94
 Velarifictorus micado WO-4 (ZJJ, 5)
 Velarifictorus micado WO-5 (ZJJ, 1)
67
73
 Velarifictorus micado WO-6 (ZJJ, 1)
 Velarifictorus micado WO-7 (ZJJ, 1)
 Velarifictorus micado WO-8 (ZJJ, 1)
 Velarifictorus micado WO-9 (ZJJ, 5)
98
99
 Velarifictorus micado WO-10 (ZJJ, 1)
 Velarifictorus micado WO-11 (ZJJ, 1)
0.020
WOVmi-1-TA
WOVmi-1-ZJJ
C
WOVmi-2-TA
WOVmi-2-ZJJ
WOVmi-3-TA
WOVmi-3-ZJJ
WOVmi-4-TA
WOVmi-5-TA
WOVmi-4-ZJJ
WOVmi-6-TA
WOVmi-5-ZJJ
WOVmi-7-TA
WOVmi-6-ZJJ
WOVmi-8-TA
WOVmi-9-TA
WOVmi-7-ZJJ
WOVmi-8-ZJJ
 Velarifictorus micado WO-1 (CS, 1)
74
 Velarifictorus micado WO-2 (CS, 1)
75
 Velarifictorus micado WO-3 (CS, 4)
 Velarifictorus micado WO-4(CS, 10)
52
 Velarifictorus micado WO-5 (CS, 1)
93
96
 Velarifictorus micado WO-6(CS, 3)
75
 Velarifictorus micado WO-7 (CS, 2)
 Velarifictorus micado WO-8 (CS, 1)
96
100
 Velarifictorus micado WO-9(CS, 1)
 Velarifictorus micado WO-10 (CS, 1)
 Velarifictorus micado WO-11(CS, 1)
81
 Velarifictorus micado WO-12 (CS, 1)
58
 Velarifictorus micado WO-13 (CS, 1)
 Velarifictorus micado WO-14 (CS, 3)
 Velarifictorus micado WO-15 (CS, 8)
70
0.020
D
 Velarifictorus micado WO-1 (LL, 3)
99
 Velarifictorus micado WO-2 (LL, 1)
94
 Velarifictorus micado WO-3 (LL, 1)
 Velarifictorus micado WO-4 (LL, 2)
 Velarifictorus micado WO-5 (LL, 2)
100
 Velarifictorus micado WO-6 (LL, 1)
0.020
B
WOVmi-1-CS
WOVmi-1-LL
WOVmi-2-CS
WOVmi-2-LL
WOVmi-3-CS
WOVmi-4-CS
WOVmi-5-CS
WOVmi-3-LL
WOVmi-6-CS
WOVmi-7-CS
WOVmi-4-LL
WOVmi-8-CS
WOVmi-9-CS
WOVmi-10-CS
WOVmi-5-LL
Supplementary Figure 1 Neighbor Joining tree for phage WO types of Velarifictorus micado-TA (A), Velarifictorus micado-CS (B), Velarifictorus micado-ZJJ (C) and Velarifictorus micado-LL (D) based on the orf7 sequences. WO-1 refers to the serial number of sequences obtained within a species. Phage WO types are shown on the right. Numbers above branches are bootstrap values computed from 1,000 replications.

## Slide 2
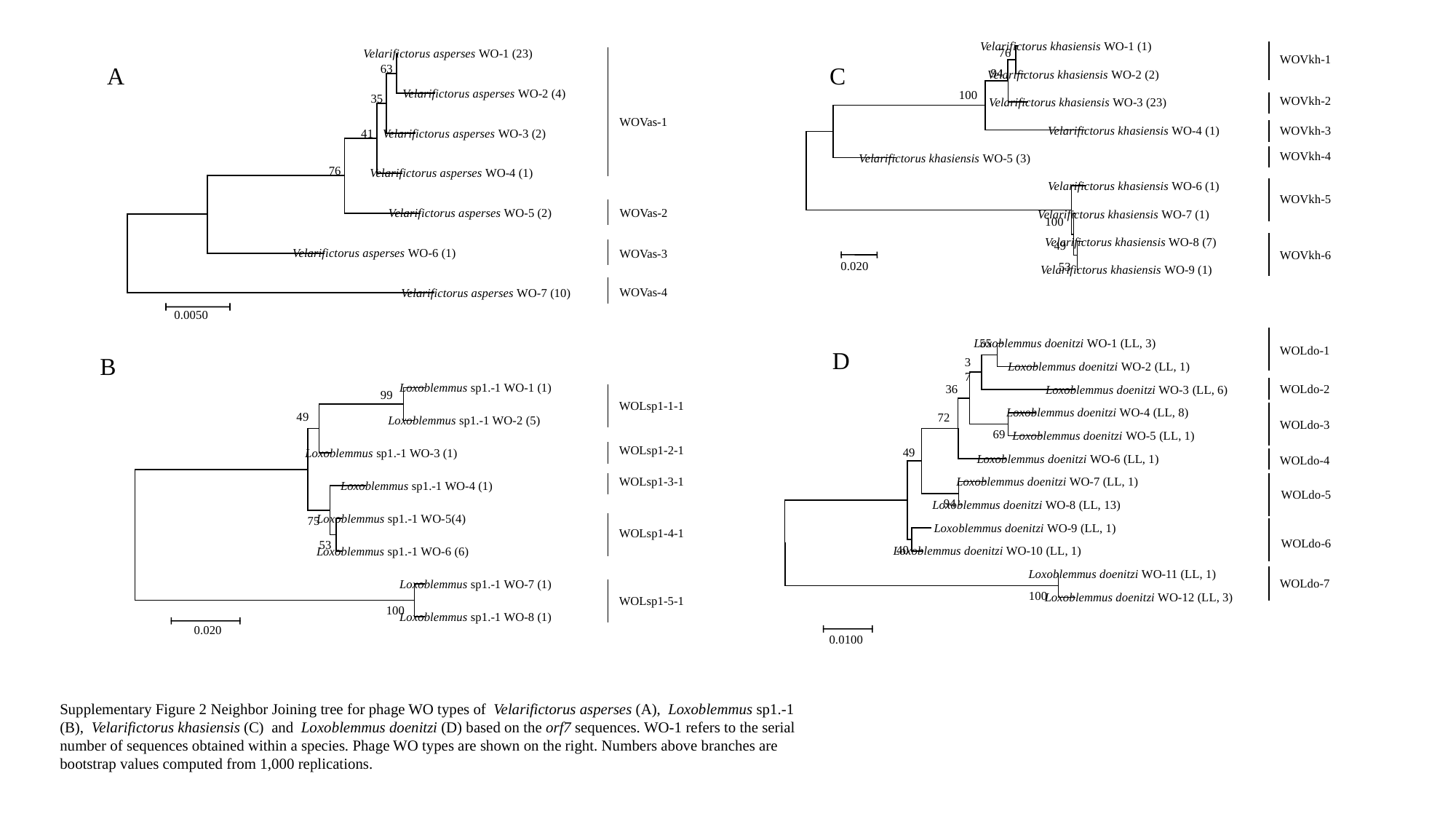

Velarifictorus khasiensis WO-1 (1)
76
94
 Velarifictorus khasiensis WO-2 (2)
100
 Velarifictorus khasiensis WO-3 (23)
 Velarifictorus khasiensis WO-4 (1)
 Velarifictorus khasiensis WO-5 (3)
 Velarifictorus khasiensis WO-6 (1)
 Velarifictorus khasiensis WO-7 (1)
100
 Velarifictorus khasiensis WO-8 (7)
49
0.020
53
 Velarifictorus khasiensis WO-9 (1)
 Velarifictorus asperses WO-1 (23)
63
 Velarifictorus asperses WO-2 (4)
35
 Velarifictorus asperses WO-3 (2)
41
76
 Velarifictorus asperses WO-4 (1)
 Velarifictorus asperses WO-5 (2)
 Velarifictorus asperses WO-6 (1)
 Velarifictorus asperses WO-7 (10)
0.0050
WOVkh-1
A
C
WOVkh-2
WOVas-1
WOVkh-3
WOVkh-4
WOVkh-5
WOVas-2
WOVas-3
WOVkh-6
WOVas-4
55
 Loxoblemmus doenitzi WO-1 (LL, 3)
37
 Loxoblemmus doenitzi WO-2 (LL, 1)
36
 Loxoblemmus doenitzi WO-3 (LL, 6)
 Loxoblemmus doenitzi WO-4 (LL, 8)
72
69
 Loxoblemmus doenitzi WO-5 (LL, 1)
49
 Loxoblemmus doenitzi WO-6 (LL, 1)
 Loxoblemmus doenitzi WO-7 (LL, 1)
94
 Loxoblemmus doenitzi WO-8 (LL, 13)
 Loxoblemmus doenitzi WO-9 (LL, 1)
40
 Loxoblemmus doenitzi WO-10 (LL, 1)
 Loxoblemmus doenitzi WO-11 (LL, 1)
100
 Loxoblemmus doenitzi WO-12 (LL, 3)
0.0100
WOLdo-1
D
B
 Loxoblemmus sp1.-1 WO-1 (1)
99
49
 Loxoblemmus sp1.-1 WO-2 (5)
 Loxoblemmus sp1.-1 WO-3 (1)
 Loxoblemmus sp1.-1 WO-4 (1)
 Loxoblemmus sp1.-1 WO-5(4)
75
53
 Loxoblemmus sp1.-1 WO-6 (6)
 Loxoblemmus sp1.-1 WO-7 (1)
100
 Loxoblemmus sp1.-1 WO-8 (1)
0.020
WOLdo-2
WOLsp1-1-1
WOLdo-3
WOLsp1-2-1
WOLdo-4
WOLsp1-3-1
WOLdo-5
WOLsp1-4-1
WOLdo-6
WOLdo-7
WOLsp1-5-1
Supplementary Figure 2 Neighbor Joining tree for phage WO types of Velarifictorus asperses (A), Loxoblemmus sp1.-1 (B), Velarifictorus khasiensis (C) and Loxoblemmus doenitzi (D) based on the orf7 sequences. WO-1 refers to the serial number of sequences obtained within a species. Phage WO types are shown on the right. Numbers above branches are bootstrap values computed from 1,000 replications.

## Slide 3
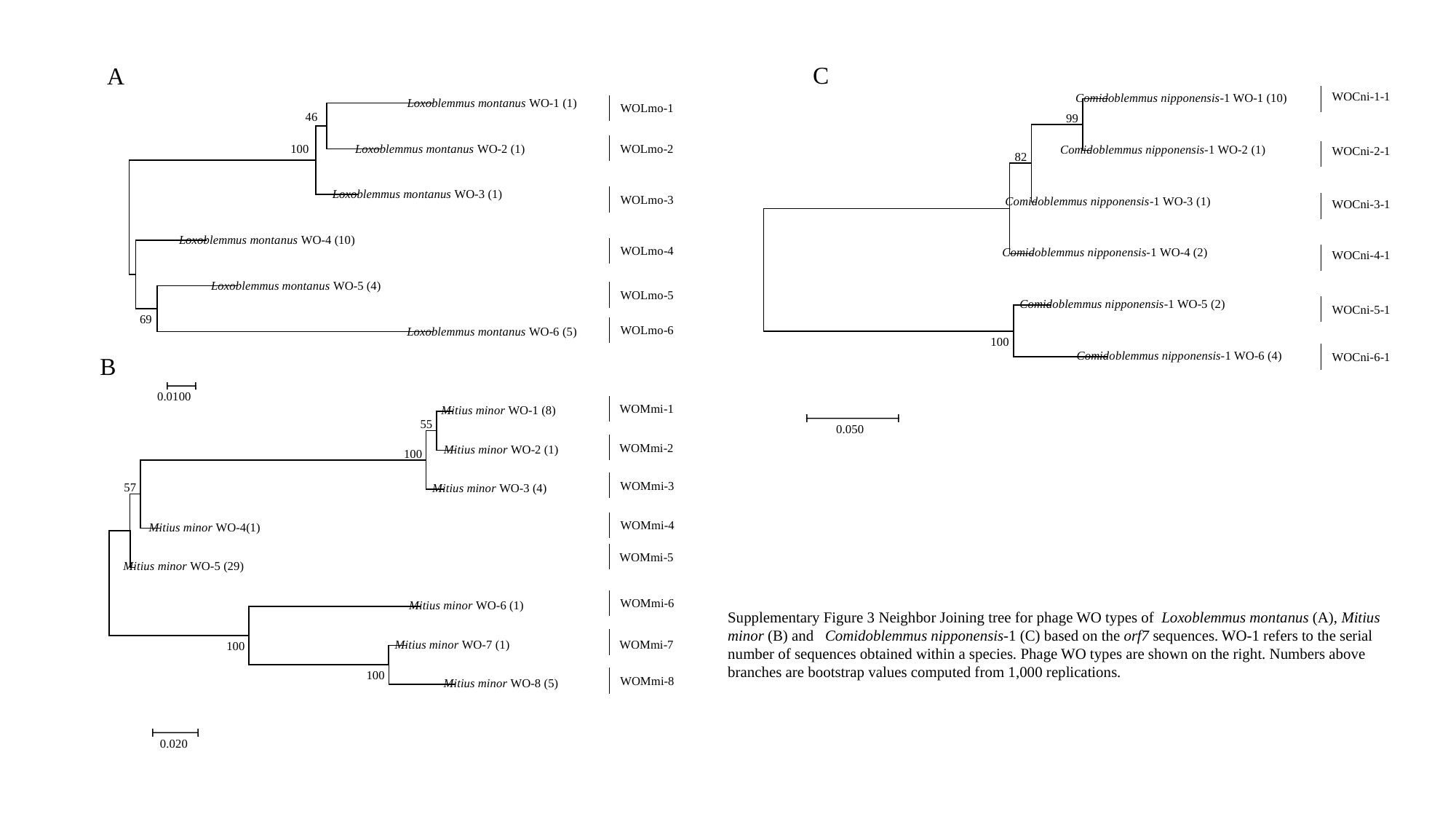

C
A
 Comidoblemmus nipponensis-1 WO-1 (10)
99
 Comidoblemmus nipponensis-1 WO-2 (1)
82
 Comidoblemmus nipponensis-1 WO-3 (1)
 Comidoblemmus nipponensis-1 WO-4 (2)
 Comidoblemmus nipponensis-1 WO-5 (2)
100
 Comidoblemmus nipponensis-1 WO-6 (4)
0.050
 Loxoblemmus montanus WO-1 (1)
46
100
 Loxoblemmus montanus WO-2 (1)
 Loxoblemmus montanus WO-3 (1)
 Loxoblemmus montanus WO-4 (10)
 Loxoblemmus montanus WO-5 (4)
69
 Loxoblemmus montanus WO-6 (5)
0.0100
WOCni-1-1
WOLmo-1
WOLmo-2
WOCni-2-1
WOLmo-3
WOCni-3-1
WOLmo-4
WOCni-4-1
WOLmo-5
WOCni-5-1
WOLmo-6
WOCni-6-1
B
 Mitius minor WO-1 (8)
55
 Mitius minor WO-2 (1)
100
57
 Mitius minor WO-3 (4)
 Mitius minor WO-4(1)
 Mitius minor WO-5 (29)
 Mitius minor WO-6 (1)
 Mitius minor WO-7 (1)
100
100
 Mitius minor WO-8 (5)
0.020
WOMmi-1
WOMmi-2
WOMmi-3
WOMmi-4
WOMmi-5
WOMmi-6
Supplementary Figure 3 Neighbor Joining tree for phage WO types of Loxoblemmus montanus (A), Mitius minor (B) and Comidoblemmus nipponensis-1 (C) based on the orf7 sequences. WO-1 refers to the serial number of sequences obtained within a species. Phage WO types are shown on the right. Numbers above branches are bootstrap values computed from 1,000 replications.
WOMmi-7
WOMmi-8
