# Supplementary material for: Phage WO diversity and evolutionary forces associated with Wolbachia-infected crickets
Source: Front Microbiol. 2025 Jan 8;15:1499315. doi: 10.3389/fmicb.2024.1499315 (PMC11750818; doi:10.3389/fmicb.2024.1499315)
Supplement: Supplementary file 3 [file Data_Sheet_1.pdf]

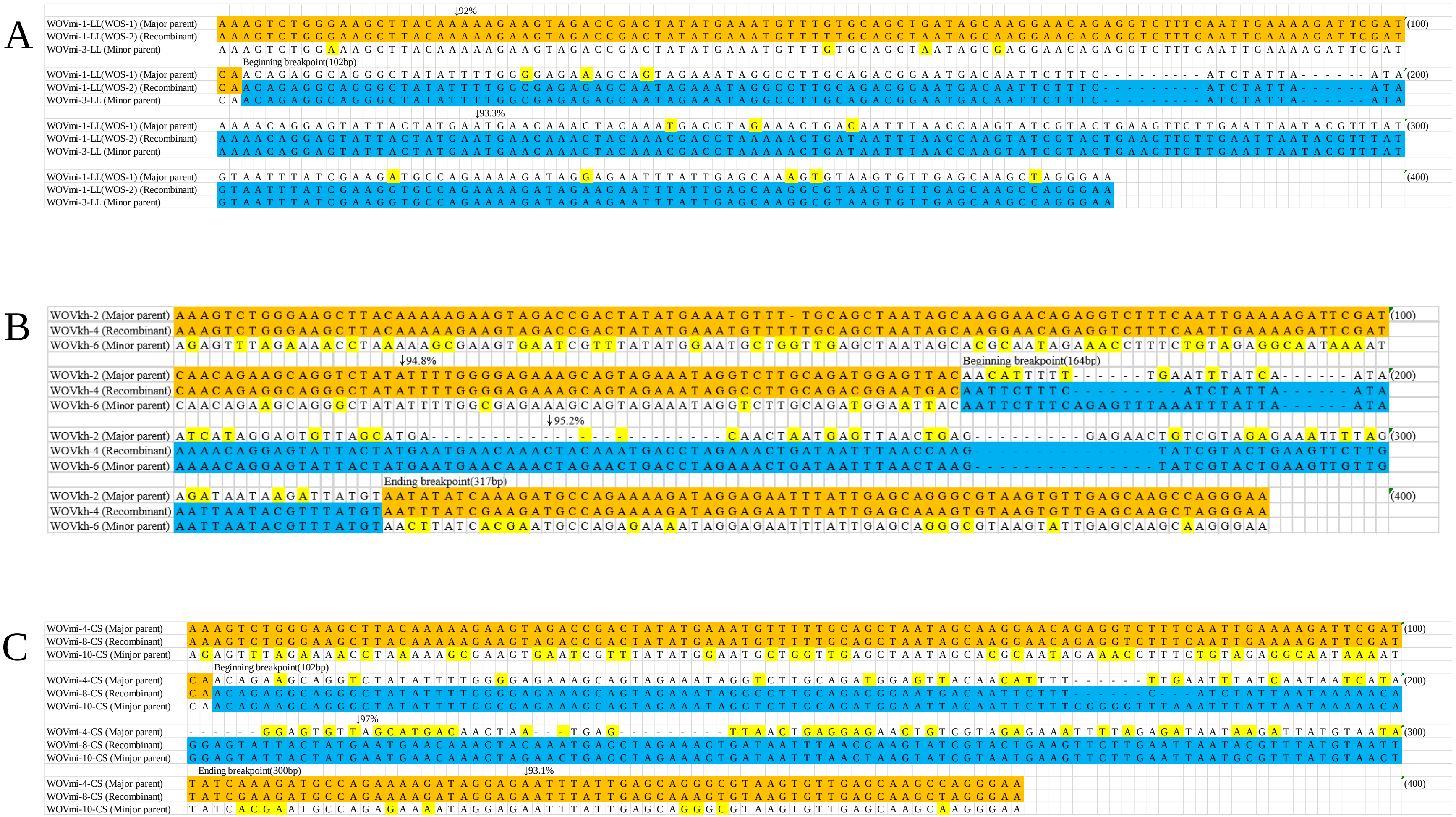

Supplementary Figure 4 Recombination events of the *orf7* gene between phage WO types WOVmi-1-LL (WOS-1) and WOVmi-3-LL resulting in recombinant WOVmi-1-LL (WOS-2) (A), WOKha-2 and WOKha-6 resulting in recombinant WOKha-4 (B), WOVmi-4-CS and WOVmi-10-CS resulting in recombinant WOVmi-8-CS (C).

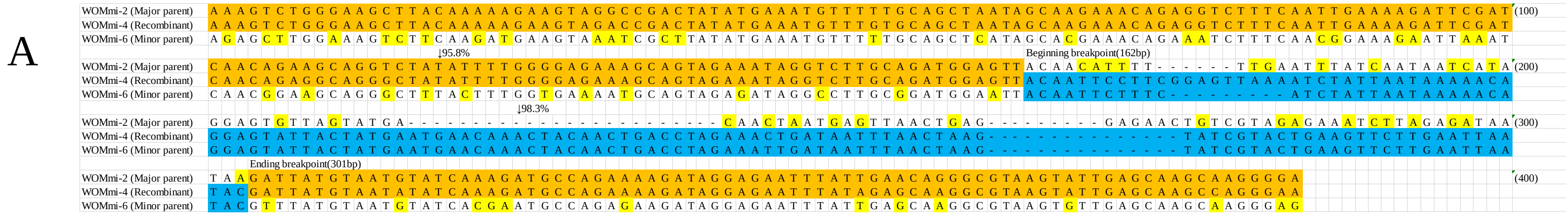

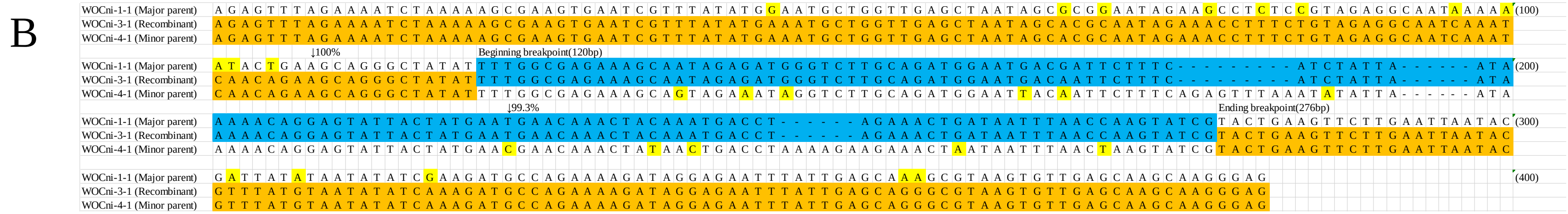

Supplementary Figure 5 Recombination events of the *orf7* gene between phage WO types WOMmi-2 and WOMmi-6 resulting in recombinant WOMmi-4 (A), WOCni-1-1 and WOCni-4-1 resulting in recombinant WOCni-3-1 (B).
